# Muscle function alterations in a Parkinson's disease animal model: Electromyographic recordings dataset

**DOI:** 10.1016/j.dib.2021.107712

**Published:** 2021-12-16

**Authors:** Ana L. Albarracín, Fernando D. Farfán

**Affiliations:** a Departamento de Bioingeniería, Facultad de Ciencias Exactas y Tecnología, Laboratorio de Investigaciones en Neurociencias y Tecnologías Aplicadas (LINTEC), Universidad Nacional de Tucumán, Tucumán, Argentina; b Instituto Superior de Investigaciones Biológicas (INSIBIO), Consejo Nacional de Investigaciones Científicas y Técnicas (CONICET), Tucumán, Argentina

**Keywords:** Parkinson's disease animal model, 6-OHDA-lesioned rats, Electromyography, Muscle dysfunction, Muscular activity in freely moving rats, Bicep femoris muscle recordings

## Abstract

Parkinson's disease (PD) is currently diagnosed based on characteristic motor dysfunctions induced by the loss of dopaminergic neurons in the substantia nigra pars compacta [1], [2]. The animal model most commonly used to reproduce PD-related motor deficits in rats is the massive degeneration of nigrostriatal neurons by using intracerebral infusion of 6-hydroxydopamine (6-OHDA) [3], [4].

This article presents data related to the research article “Quantifying muscle alterations in a Parkinson's disease animal model using electromyographic biomarkers” [5]. This study evaluated the effect of PD neurotoxic lesion model on muscle function of freely moving rats. The effects on muscle function considering the time post-lesion have never been described for this Parkinson's disease model. Electromyographic recordings were obtained from control and hemiparkinsonian rats walking in a circular treadmill. Chronic EMG electrodes were implanted subcutaneously in a hindlimb muscle - the biceps femoris muscle - for evaluating muscular activity during the gait. Five dataset of EMG recordings are presented in this article corresponding to control animals and four groups of lesioned animals at different time post-injury (three to six weeks after lesion). Stationarity of the EMG signals were established and the effective muscular contractions were detected by using signal processing methods described in [5]. Power spectrum density was characterized through the mean and median frequencies and signals probability distribution function analysis was also performed.These analyses have shown that PSD frequency contents progressively fall with time post-lesion suggesting muscle function changes along this enclosed time.

This dataset could be reused to investigate muscular activation parameters under control and lesioned conditions in freely moving rats and for evaluating different signal processing methods for EMG patterns detection.

## Specifications Table

**Table:** 

Subject	Neuroscience, Neurophysiology, Signal processing
Specific subject area	Parkinson's disease animal model, Electromyography
Type of data	*.txt
How data were acquired	EMG signals were acquired (2000 sampling rate), amplified (60dB) and filtered (50 Hz notch filter and 30–500 Hz bandpass) through Biopac System and the software BSL (www.biopac.com).
Data format	Raw
Description of data collection	24 male adult Wistar rats were used for this study. They were divided in 5 groups: one control group (N=5) and four groups of hemiparkinsonian rats with different time post-lesion (N=19). EMG recordings were obtained by using an electrode array subcutaneously implanted in the bicep femoris muscle of rats left hindlimb. EMG signals were collected after 3-, 4-, 5- and 6-weeks post-lesion to analyse possible changes in the muscle function. In general, it was able to obtain EMG signals during 3 consecutive days but on occasions only 2; in the latter case, it was verified the formation of scar tissue around the electrodes that may interfere with the signal recordings.
Parameters for data collection	Rats were placed on the circular treadmill so that the left hind paw – implanted - was out of the circular path. Each recording lasted at least 2 min in order to collect enough contractions from the muscle for an accurate analysis. EMG signals were acquired (2000 sampling rate), amplified (60dB) and filtered (50 Hz notch filter and 30–500 Hz bandpass) through Biopac System
Data accessibility	https://data.mendeley.com/datasets/vpm6gdt4np/2 DOI:10.17632/vpm6gdt4np.2[6]
Related research article	Teruya Pablo Y, Farfán Fernando D, Pizá Álvaro G, Soletta Jorge H, Lucianna Facundo A and Albarracín Ana L. Quantifying muscle alterations in a Parkinson's disease animal model using electromyographic biomarkers. Medical & Biological Engineering & Computing. DOI:10.1007/s11517-021-02400-3

## Value of the Data

• This dataset contributes to understanding the effect of the Parkinson's disease lesion model with 6-hidroxidopamine in the muscular function. Motor deficits are the cardinal symptoms in the early diagnosis of the illness. Motor deficits characterization in this model is crucial for the comprehension of the related central motor dysfunction.
• EMG signals were recorded with a reliable chronic electrode and obtained from freely moving animals. Investigations using this experimental design are benefited from a realistic condition for studying the effects of the lesion and the motor deficits. Researchers could take advantage of these data to establish possible correlations with previous experimental approaches in the field.
• EMG signals obtained with the experimental conditions we have proposed (*in vivo* and subcutaneous recordings), are excellent conditions to evaluate different signal processing techniques. It could be very useful for evaluating techniques for removing noise or for detecting different signal patterns.
• This data provides an electrophysiological evaluation of muscle function in this PD model along the time post-lesion. It could be useful for researchers working in the same PD model to compare our electrophysiological results with experimental results in gait analysis, behavioural tests, neurochemical determinations or other evaluations in this model of Parkinson's disease.

## 1 Data Description

The data is included in two folders:

(1) CONTROLS: contains raw EMG recordings collected from control animals
(2) LESIONED: contains raw EMG recordings collected from lesioned or experimental animals. It includes recordings from three to six weeks post-lesioned animals.

### 1.1 File's name structure

The data provided in this article are raw EMGs recordings exported from the Biopac System. The files were named considering: (1) the animal number, (2) the group each animal belongs to, (3) the time post-lesion, in the case of lesioned animals, (4) trial day number and (5) the trial.

### 1.2 Examples of file's name

1)  CONTROLS folder

C-1-1A

C= control animal (not lesioned)

1= animal number

1A= trial number; 1: first recording day; A: first trial

2) LESIONED folder

L3-6-2B

L= lesioned; 3: week 3, post-lesioned time

6= animal number

2B= trial number; 2: second recording day; B: second trial

### 1.3 Details about recordings

Table 1 enumerates the EMG recordings collected from control and lesioned rats. Five control animals (rats 1–5) were evaluated; four recordings were obtained from rats 1 to 4 during two consecutive days; two recordings were obtained from rat 5 in one day. In addition, it was obtained at least two recordings from each lesioned animal; in the best case, it was obtained six recordings from one rat along three consecutive days (for instance, in the case of rats 14 and 18).

**Table 1:** List of recordings obtained from control and lesioned rats.

Control rats				
*Rat 1*	*Rat 2*	*Rat 3*	*Rat 4*	*Rat 5*
C-1-1A C-1-1B C-1-2A C-1-2B	C-2-1A C-2-1B C-2-2A C-2-2B	C-3-1A C-3-1B C-3-2A C-3-2B	C-4-1A C-4-1B C-4-2A C-4-2B	C-5-1A C-5-1B

Lesioned rats – 3 weeks post-lesion				

*Rat 6*	*Rat 7*	*Rat 8*	*Rat 9*	*Rat 10*

L3-6-1A L3-6-1B L3-6-2A L3-6-2B	L3-7-1A L3-7-1B L3-7-2A L3-7-2B	L3-8-1A L3-8-1B L3-8-2A L3 -8-2B	L3-9-1A L3-9-1B L3-9-2A L3-9-2B	L3-10-1A L3-10-1B L3-10-2A L3-10-2B

Lesioned rats – 4 weeks post-lesion				

*Rat 11*	*Rat 12*	*Rat 13*	*Rat 14*	*Rats 15 and 16*

L4-11-1A L4-11-1B L4-11-2A L4-11-2B	L4-12-1A L4-12-1B	L4-13-1A L4-13-1B L4-13-2A L4-13-2B	L4-14-1A L4-14-1B L4-14-2A L4-14-2B L4-14-3A L4-14-3B	L4-15-1A L4-15-1B L4-15-2A L4-15-2B L4-16-1A L4-16-1B

Lesioned rats – 5 weeks post-lesion				

*Rat 17*	*Rat 18*	*Rat 19*	*Rat 20*	*Rat 21*

L5-17-1A L5-17-1B L5-17-2A L5-17-2B	L5-18-1A L5-18-1B L5-18-2A L5-18-2B L5-18-3A L5-18-3B	L5-19-1A L5-19-1B L5-19-2A L5-19-2B	L5-20-1A L5-20-1B L5-20-2A	L5-21-1A L5-21-1B

Lesioned rats – 6 weeks post-lesion				

*Rat 22*	*Rat 23*	*Rat 24*	*Rat 25*	

L6-22-1A L6-22-1B L6-22-2A L6-22-2B	L6-23-1A L6-23-1B L6-23-2A L6-23-2B	L6-24-1A L6-24-1B	L6-25-1A L6-25-1B	

## 2 Experimental Design, Materials and Methods

The data included in this article belongs to an experimental study focused on evaluating muscle function of rats affected by a neurotoxic PD model along time post-lesion [5].

The experiments consisted in recording the biceps femoris muscle activity of the rat's hindlimb. The biceps femoris is the largest muscle in the hindlimb and its principal functions include thigh abduction, hip extension and knee flexion. In order to evaluate muscle dysfunction, EMG signals were obtained from freely moving animals, after a post-surgery period required for animal recovery.

Twenty-four male adult Wistar rats were used for this study. The decision to use males for the experiments is related to the hypothesis that suggests that estrogens may exert beneficial effects against the development and the progression of the disease [7].

Five animals were used as controls and nineteen rats underwent a surgery protocol for a neurotoxic Parkinson's disease model. The model consisted in the use of 6-hidroxidopamine (6-OHDA) as a toxin to lesion mesencephalic dopaminergic neurons and generate a unilateral model of the disease. The lesions were made on the right hemisphere and, as the motor pathways to the hindlimbs are contralateral, the hindlimb affected by the lesion were the left one. Therefore, we have obtained the EMG recordings from the left hindlimb biceps femoris muscle of the hemiparkinsonian rats. The effectiveness of the lesions was corroborated by the apomorphine-induced rotation test after an appropriate time post-injection of the toxin into the rat brain(three weeks).

A circular treadmill previously built was modified to allow electrophysiological recordings of freely moving rodents [8]. This device consists of a transparent circular structure made of glass floor and acetate wall (20 cm high). To limit the rat displacements, an inner circular wall of plastic cardboard was added, thus leaving an effective walking space of 8 cm wide.

On the other hand, an EMG electrodes array was designed for recording the activity of small muscles from the rat hindlimbs. Electrodes were made of stainless steel and the contact surface was around 2.6 mm^2^. Two electrodes were used in a bipolar configuration for EMG recordings and one wire served as a ground electrode.

The surgical procedure for the chronical electrode implantation was made under general anaesthesia with ketamine and xylazine. The electrode was located on the biceps femoris muscle surface and it was sutured to the fascia of the muscle in order to get a good fixation. This method prevents the damage of the muscle fibers and also allows the correct detection of the signal from the muscle surface. The wires were conducted subcutaneously to reach a 3-pin socket attached to the surface of the skull by dental cement.

**Fig. 1:**
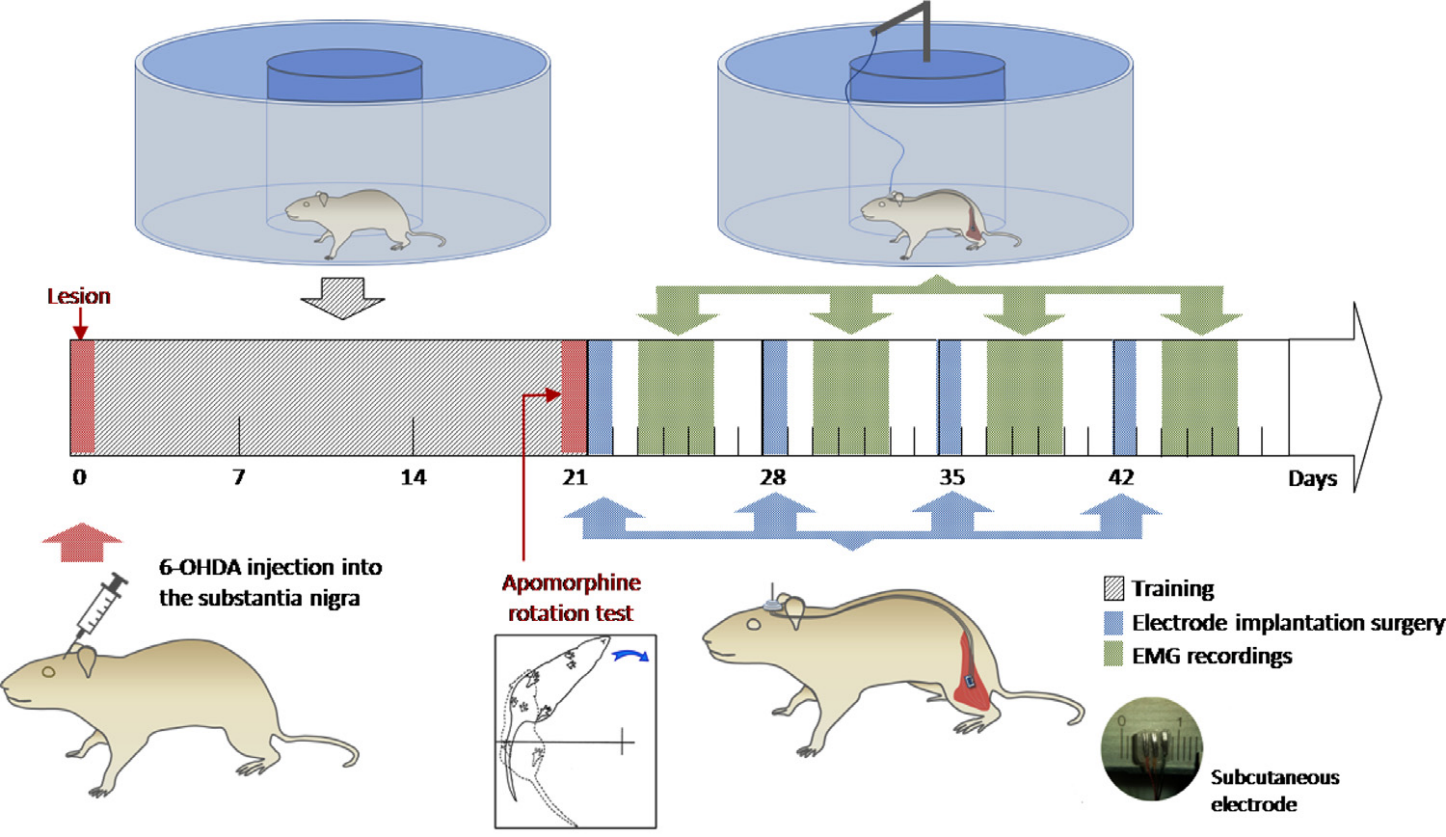
Diagram of the experimental protocol used for collecting the data.

After a time for recovery, rats were placed on the treadmill for data collection. Each recording lasted at least 2 min in order to collect enough contractions from the muscle for an accurate analysis. Fig. 1 shows a diagram of the experimental protocol.

The sites of electrode implantations were verified once the recordings were completed after the euthanasia of each animal. It was verified for all animals that electrodes were exactly on the implantation site previously fixed. In general, we were able to obtain EMG signals during 3 consecutive days but in occasions only 2 (Table 1); in these cases, it was verified the formation of scar tissue between muscle surface and electrodes that would interfere with the signal recordings.

### 2.1 EMG recordings samples

**Fig. 2:**
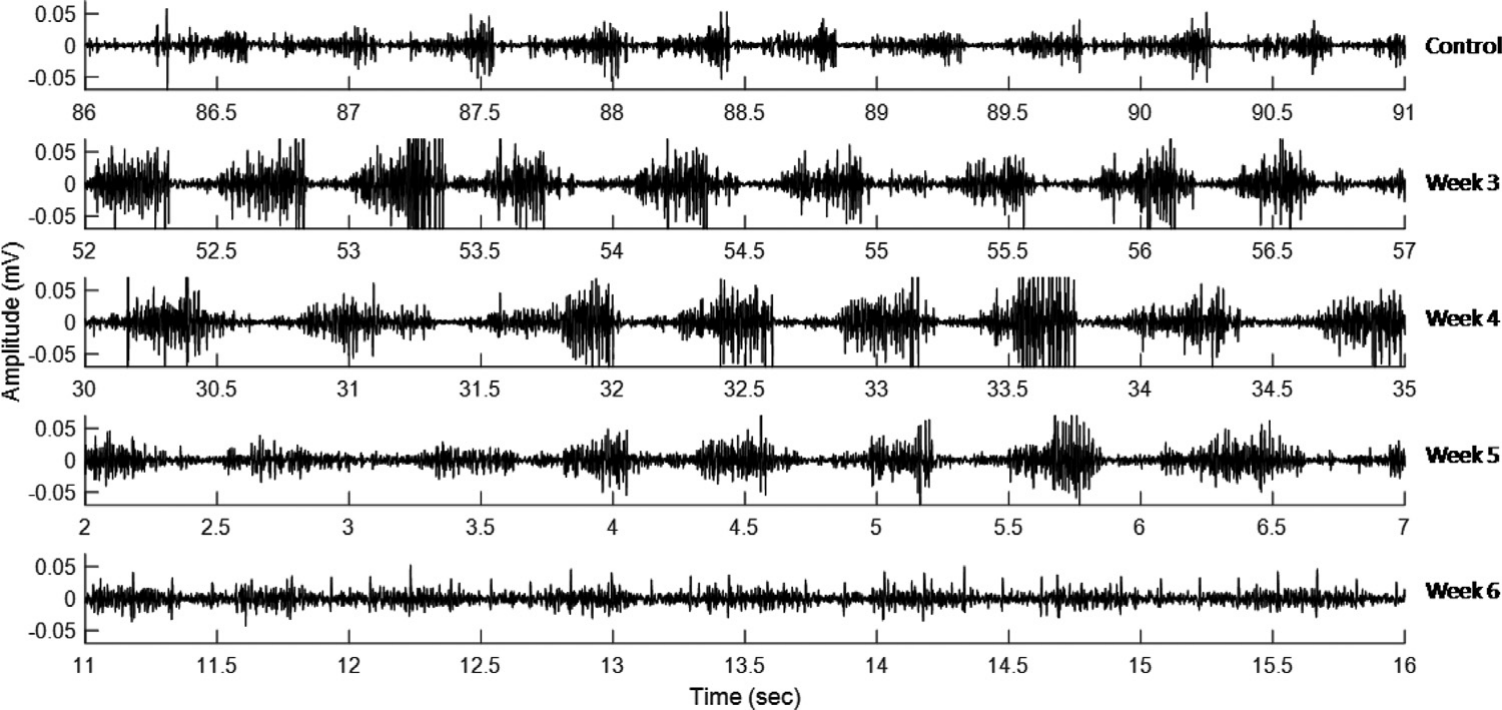
EMG recording samples. From top to bottom, recordings obtained from a control rat (file 'C-1-2B.txt') and from hemiparkinsonian rats: with 3 weeks post-lesion (file 'L3-6-2A.txt'), 4 weeks post-lesion (file 'L4-14-1B.txt'), 5 weeks post-lesion (file 'L5-17-2A.txt'), and 6 weeks post-lesion (file 'L6-24-1B.txt').

As examples of the data enclosed with this work, Fig. 2 shows EMG recordings samples obtained from the biceps femoris muscle of control and hemiparkinsonian rats.

### 2.2 Steps summary to obtain the data

1) Select 24 male wistar adult rats and divide them in two groups:
  • control rats or not lesioned (N=5)
  • rats that will be lesioned with 6-OHDA (N=19)
2) Divide the lesioned group into four groups considering the time post-lesion the recordings will be obtained:
  • week 3 post-lesioned (N=5)
  • week 4 post-lesioned (N=5)
  • week 5 post-lesioned (N=5)
  • week 6 post-lesioned (N=4)
3) Lesion the animals previously selected with 6-OHDA injections into the substantia nigra, following the methodology described above. This procedure must be made the same day for all animals in order to obtain the same experimental conditions.
4) After a time for recovery from the lesion surgery, start the training of the animals to walk on the treadmill. The training must be done for three weeks and one time each day.
5) Three weeks after the 6-OHDA-lesion surgery, animals must be tested for the effectiveness of the lesion injecting subcutaneously with apomorphine (0.25 mg/kg) for testing the rotational behavior contralateral to the lesioned side.
6) Once the lesion was corroborated in the animals, they must be submitted to the electrode implantation surgery. Control animals also must receive the same implantation surgery.
7) After a time for recovery from the surgery, animals can be placed on the treadmill to obtain the recordings from the biceps femoris muscle
8) Each recording must last 2 min at least in order to collect enough contractions from the muscle for an accurate analysis.

## Ethics Statement

The dataset included in this article was collected from adult male rats belonging to the Bioterium of the Medicine Faculty, National University of Tucumán. All procedures described here were performed in accordance with the National Institute of Health guide for the Care and Use of Laboratory Animals (NIH Publications No. 8023, revised 1978) and Guidelines for the Use of Animals in Biomedical Research. The protocol was also approved by the local bioethics committee (Resol.41/2015, Medicine Faculty, National University of Tucumán). All experiments were performed under ketamine/xylazine anaesthesia, and every effort was made to minimize animal suffering. Supplemental doses of anaesthesia were used over the course of the surgical procedure to maintain an appropriate plane of anaesthesia.

## CRediT authorship contribution statement

**Ana L. Albarracín:** Conceptualization, Methodology, Writing – review & editing. **Fernando D. Farfán:** Data curation, Methodology, Software, Writing – original draft.

## Declaration of Competing Interest

The authors declare that they have no known competing financial interests or personal relationships which have or could be perceived to have influenced the work reported in this article.
